# Dual-Energy Computed Tomography of the Liver: Uses in Clinical Practices and Applications

**DOI:** 10.3390/diagnostics11020161

**Published:** 2021-01-22

**Authors:** Masakatsu Tsurusaki, Keitaro Sofue, Masatoshi Hori, Kosuke Sasaki, Kazunari Ishii, Takamichi Murakami, Masatoshi Kudo

**Affiliations:** 1 Department of Radiology, Faculty of Medicine, Kindai University, Osakasayama 589-8511, Japan; ishii@med.kindai.ac.jp; 2 Department of Radiology, Graduate School of Medicine, Kobe University, Kobe 650-0017, Japan; keitarosofue@yahoo.co.jp (K.S.); horimsts@med.kobe-u.ac.jp (M.H.); murataka@med.kobe-u.ac.jp (T.M.); 3 CT Research Group, GE Healthcare Japan, Hino 191-8503, Japan; kosuke.sasaki@ge.com; 4 Department of Gastroenterology and Hepatology, Kindai University, Faculty of Medicine, Osakasayama 589-8511, Japan; m-kudo@med.kindai.ac.jp

**Keywords:** computed tomography, dual-energy CT, imaging, hepatic steatosis, hepatic fibrosis, cirrhosis

## Abstract

Dual-energy computed tomography (DECT) is an imaging technique based on data acquisition at two different energy settings. Recent advances in CT have allowed data acquisitions and simultaneous analyses of X-rays at two energy levels, and have resulted in novel developments in the field of abdominal imaging. The use of low and high X-ray tube voltages in DECT provide fused images that improve the detection of liver tumors owing to the higher contrast-to-noise ratio (CNR) of the tumor compared with the liver. The use of contrast agents in CT scanning improves image quality by enhancing the CNR and signal-to-noise ratio while reducing beam-hardening artifacts. DECT can improve detection and characterization of hepatic abnormalities, including mass lesions. The technique can also be used for the diagnosis of steatosis and iron overload. This article reviews and illustrates the different applications of DECT in liver imaging.

## 1. Introduction

Computed tomography (CT) is one of the most important diagnostic imaging modalities for the liver. Technical advances such as faster scan times, thinner slices, multiplanar reformatting, and three-dimensional (3D) rendering have revolutionized the utility of CT. DECT is an exciting recent development that promises to increase the modality’s potential [1,2]. This improvement derives from the exploitation of the energy-dependent attenuation of X-rays at two different energies, hence, justifying the term dual energy. Conventional X-ray CT produces images based on the detection of the energy-dependent attenuation of X-rays as they pass through scanned objects. DECT extends this core concept based on the use of X-rays generated at two different energies. This resolves X-ray attenuation ambiguities and therefore allows for superior material discrimination and characterization. DECT refers to the acquisition of CT datasets at two different energy spectra. Paramount among the challenges of early DECT systems was the fact that the dual-energy data had to be obtained with two separate scans that resulted in multiple complications, such as the misregistration of the two datasets [3,4,5,6,7,8]. The technical advancements in software and hardware ion mid- to late 2000s allowed the simultaneous generation and acquisition of spectral X-rays. The recent development of dual-energy scanning increased the diagnostic value and clinical applications of CT and made DECT feasible for routine clinical use.

In this article, we will explore DECT using fast kVp switching and its application to liver imaging. First, an overview of the basic principles and physics of DECT imaging and image reconstruction is given. Subsequently, the clinical utility and benefits of DECT using fast kVp switching are described, including accurate material quantification, improved detection and characterization of liver lesions, and reduced iodine and radiation doses. We conclude that the basic knowledge and clinical merits of DECT need to be translated into routine clinical practice for improved patient care.

## 2. Recent Developments and Basic Principles of DECT

DECT was first conceptualized in the 1970s [3]. Various methods have been studied in various fields for CT diagnosis. Various methods have been introduced. These included photon-counting methods [3,4], such as dual-photon absorptiometry, the sandwich detector method using a multilayer detector [5,6], and kV switching methods [7,8], such as the dual-energy X-Ray absorptiometry. However, the clinical application of DECT has only recently been realized as a result of robust improvements in performance and postprocessing capabilities. Conventional or single-energy CT (SECT) utilizes a single polychromatic X-ray beam (ranging from 70 to 140 kVp, typically at 120 kVp) emitted from a single source and received by a single detector. The inherent contrast of the image dataset generated by this process depends on differences in photon attenuation of the various materials that constitute the human body (i.e., soft tissue, air, calcium, fat). The degree that a material will attenuate the X-ray beam is dependent on tissue composition and photon energy level, and how closely it exceeds the K-edge (i.e., inner electron shell binding energy) of the material. Therefore, tissue attenuation can be manipulated by changing photon energy levels. In DECT, two energy levels (typically 80 and 140 kVp) are used to acquire images that can be processed to generate additional datasets.

**Figure 1:**
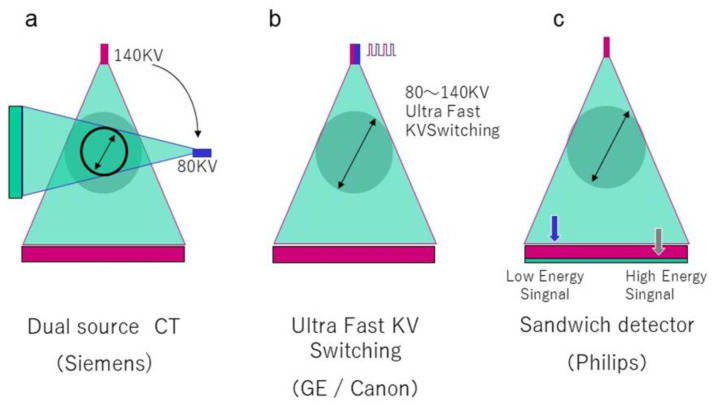
Imaging Method of Dual-Energy Computed Tomography (CT). This is a schematic showing (**a**) a dual-source CT in which X-rays of different tube voltages are simultaneously irradiated from two bulbs with 90° orientation differences, (**b**) a single-source CT system in which the tube voltage is switched to a fast switching mode (Fast kV switching) of 0.5 ms or less, and (**c**) a single-tube voltage X-ray irradiated with a stacked detector.

There are currently some DECT platforms marketed by major CT vendors. Dual-source DECT (dsDECT) (Somatom Drive/Somatom Definition Flash, Siemens Medical Solutions, Forchheim, Germany) utilizes two X-ray tubes and two detectors to obtain simultaneous dual-energy acquisitions and data processing. Single-source DECT (ssDECT) (Revolution CT, GE Healthcare, Milwaukee, WI, USA), (Aquilion ONE GENESIS Edition, Canon Medical Systems, Otawara, Japan) uses a single X-ray tube that rapidly alternates between low and high energies (fast switching), and a single detector that quickly registers information from both energies. In detector-based spectral CT (IQon spectral CT, Philips Healthcare, Eindhoven, The Netherlands), a single X-ray tube with full-dose modulation capabilities is paired with a detector made of two layers (sandwich detector) that simultaneously detects two energy levels (Figure 1). It is important to aware each technique’s strengths and limitations. At first, dsDECT technique provides that tube current modulation is possible, but the dual-energy field of view (DE-FOV) is limited, particularly in earlier generations. Due to a limited DE-FOV centering of patient within the gantry according to the organ of interest, in this case the liver becomes critical. Fink et al. suggested the use of a thick collimation and centering the patient to the left in the first-generation dsDECT to compensate for the restricted FOV (26 cm) [9,10]. Second- and third-generation dsDECT scanners have a tin filter at the high-energy tube output allowing for better spectral separation. These scanners also provide a larger DE-FOV and better spatial resolution due to the use of thinner collimation. In contrast, while tube current modulation is impossible in fast-switching ssDECT, but fast-switching ssDECT technique provides a larger effective DE-FOV; 50 cm.

There is no previous report that has described the comparison of the radiation dose among these vendors. Although two energies are used in DECT, the radiation dose is not twice the dose of conventional CT when fast switching (i.e., ssDECT) is performed, because the dwell time is longer at the lower energy than at the higher energy. The dose length product was reduced by 0.3–20.1% in ssDECT (gemstone spectral imaging (GSI) with rapid kV switching technique) compared to conventional imaging [11]. Although initial dsDECT revealed a three times higher radiation dose than single-energy CT, recent studies have demonstrated that third-generation dsDECTs can be performed without a radiation dose penalty or impairment of image quality compared to single-energy CT with 100–120 kVp [12,13]. On the other hands, the necessity to increase the tube potential to at least 120 kVp for spectral imaging with current dual-layer detector (DL) CT results in an increase of patient radiation exposure, compared with conventional single-layer acquisitions performed at 100 kVp. However, dual-layer detector (DL) CT at 40–45 keV by adjusting automated exposure control (i.e., DRI) allows for a 20% reduction in radiation dose, while preserving image quality comparable to that of conventional 120 kVp protocol [14].

**Figure 2:**
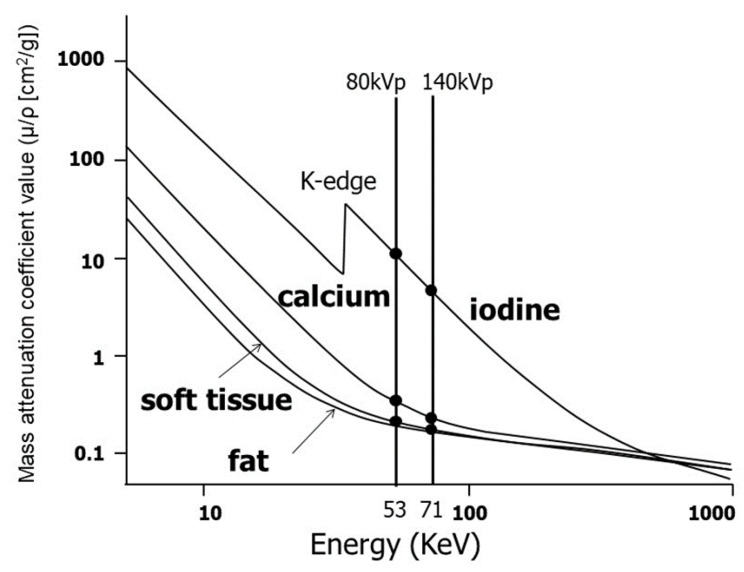
Mass attenuation coefficient values (μ/ρ [cm^2^/g]) of four materials commonly observed in diagnostic dual-energy CT (DECT) imaging as a function of X-ray energy (keV, logarithmic scales are used on both the horizontal and vertical axes). At lower energy, the mass attenuation coefficients of low-effective atomic number (*Z*) materials, such as fat (*Z* = 6, 8) and soft tissue (*Z* = 1, 8) is moderately elevated, whereas those of high Z materials, such as bone and calcification consisting of Ca (*Z* = 20) and iodine (*Z* = 53) increase abruptly. Note that the distinctive K-edge of iodine results from increased attenuation of X-rays by photoelectric absorption at 33 keV, which is equal to electron-binding energies for the K shell. The available range of virtual monochromatic energy (VME) images that can be produced by fast kVp switching DECT is also shown.

The governing principle of CT is the dependence of the mass attenuation coefficient of a given material on the energy of the incident X-rays (Figure 2). The mass attenuation coefficient is defined as *μ/ρ* (cm^2^/g) in which *μ* (cm^−1^) is the linear attenuation coefficient, *ρ* (g/cm^3^) is the density of the material, and the X-ray energy is measured in kiloelectron volts (keV). An important consideration regarding the relationship between X-ray attenuation and material density is that the same attenuation value can be obtained for two different materials at a given energy depending upon the material densities. For example, bone (*ρ* ≈ 1 g/cm^3^) and iodine (*ρ* ≈ 0.1 g/cm^3^) have the same linear attenuation coefficient at approximately 100 keV [2].

X-ray energy is commonly referred to in terms of kVp and keV. The more common polychromatic energy beam used in conventional multidetector CT (MDCT) scanners is kVp (the maximum energy in polychromatic spectra, i.e., 140 kVp, thus indicating a spread of photon energies, with 140 kVp being the highest), and keV representing the monochromatic beams (by definition, a monochromatic beam contains only a single energy (i.e., 70 keV represents the single energy present within that beam). Therefore, the Hounsfield units (HU) of any material can be plotted on a curve as a function of kVp or keV. Furthermore, the density curve for any material can be represented by the combination of the known curves of two other materials. These other materials are known as basis materials. Two of the most common used basis materials in clinical settings are iodine and water in view of fact that they are sufficiently different in atomic numbers as to represent the range of densities commonly used in medical imaging.

The upper limit of the energy spectrum produced by an X-ray tube is determined by the peak tube voltage (kVp) [15]. With conventional (single energy) CT, the energy level is typically set to 120 or 140 kVp. With DECT, the energy settings are typically 80 kVp and140 kVp, thus creating low- and high-energy datasets, respectively. The inherent energy of an X-ray photon and the atomic number of the interacting matter will impact the degree of attenuation. A spike in attenuation, termed the K-edge, occurs when the X-ray energy level is just greater than the electron-binding energy of the interacting matter [16,17]. K-edge differs for each element and increases as a function of the atomic number. DECT capitalizes on the variable K-edges and on the differences in attenuation among tissues at different X-ray energies [18,19]. Therefore, at 80 kVp, more photons will be near 33.2 keV (the K-edge of iodine) than at 140 kVp [20]. By applying these concepts, DECT can provide information about tissue composition and behavior at different energy levels. It can differentiate between tissues that contain different substances (e.g., iodine at 33.2 keV or calcium at 4.0 keV versus other soft tissues) because certain substances have higher attenuation at lower kVp settings. Furthermore, by using some postprocessing techniques, the change in attenuation at different kVp values after intravenous administration of iodinated contrast material can be used to create a virtual unenhanced dataset by excluding iodine-containing pixels [16,17]. The applications of abdominal DECT vary by organ system, as outlined below.

## 3. Information Obtained through Dual-Energy Imaging and Its Clinical Applications

All data described in this manuscript were acquired with a single-source, 64-channel multidetector CT with fast kVp switching (Discovery CT750 HD, GE Healthcare, Milwaukee, WI, USA) at a single institution. The principle of operation of the Discovery CT750 HD GSI (Gemstone Spectral Imaging) is based on fast KV switching between two different energy levels of X-rays on adjacent views during a single rotation. Both high- and low-energy datasets were acquired simultaneously for axial and helical acquisitions at the full field-of-view of 50 cm. This is enabled by a generator that can switch reliably between 80 and 140 kVp targets in less than 0.5 ms.

**Figure 3:**
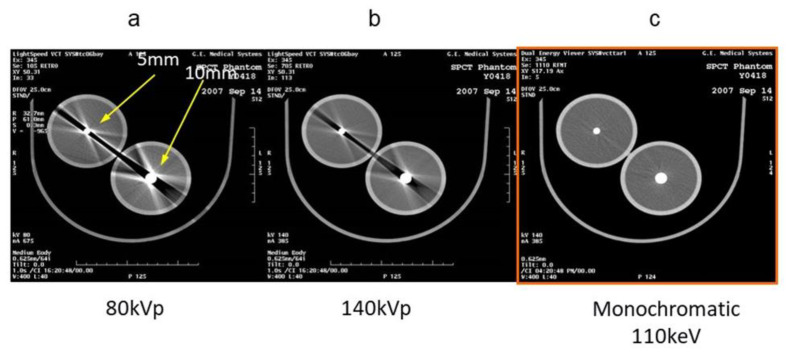
Beam-hardening artifact of the metal phantom using conventional multicolor X-ray CT imaging and virtual monochromatic imaging. Conventional polychromatic X-ray CT images (**a**,**b**), and tube voltages are 80 and 140 KVp. Beam-hardening artifacts derived from metal are observed especially at low-tube currents. (**c**) Virtual monochromatic image at 110 keV on dual-energy CT image, beam-hardening artifact, and CT value around metal are suppressed.

This method achieves all the proper features of dual energy: good energy resolution, low temporal and spatial misregistration between the two energies, and no limitation in the radiographic field. The dual-energy image reconstruction of GE performs beam-hardening correction based on water and iodine substances on the projection data [21]. In addition, the image calculated from this data has greatly improved beam-hardening compared with conventional CT (Figure 3), and has thus allowed dual-energy imaging within the entire-body region. In low-keV images, contrast enhancement can potentially reduce the volume of contrast medium, and can enhance the visualization of peripheral blood vessels and mass lesions. Beam-hardening artifacts owing to metals can be reduced in high-keV images. In addition to the general dual-energy application [21,22], there are numerous research reports that have used various density values, such as iodine and fat, and effective atomic number analysis. Mileto et al. reported that lipid-poor adenomas and nonadenomas that could not be distinguished by conventional CT were successfully distinguished by DECT using the density values of iodine, fat, and water [23]. Shinohara et al. suggested the possibility of characterizing cervical plaques using an effective atomic number [24], and Aoki et al. reported that iodine density values were more useful than CT values in predicting recurrence after trunk stereotactic radiotherapy for lung tumors [25].

There are two pieces of information that can be obtained by performing imaging using different tube voltages, namely “virtual monochromatic images” and “material decomposition”.

### 3.1. Virtual Monochromatic Imaging

**Figure 4:**
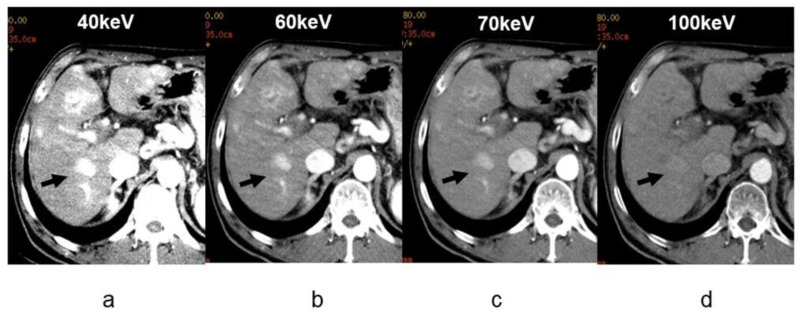
Contrast enhancement of virtual monochromatic images in the same patient. A dynamic CT arterial phase at (**a**) 40, (**b**) 60, (**c**) 70, and (**d**) 100 keV. The 120 kVp CT used in conventional CT is approximately equivalent to the 70 keV image. The difference in contrast enhancement in the deep staining (arrow) of the hepatocellular carcinoma induced by the differences in keV is clear. The contrast enhancement at low-keV values is high but noisy, while the enhancement at high keV such as 100 KeV is clearly poor.

In DECT, an image can be produced in a pseudo manner by monochromatic X-ray from 40 keV to 140 keV. This is called a virtual monochromatic image (hereinafter monochromatic image). The DECT device with the fast kV switching method that we use at our hospital can provide a monochromatic image at 1 keV increments between 40 keV and 140 keV. Dynamic multiphasic contrast CT is usually performed in liver imaging. In virtual monochromatic X-ray imaging, images with good contrast can be obtained easily by selecting the best keV setting (Figure 4). In other words, if an image of keV with higher iodine CT value is used for the arterial phase image, contrast is expected to increase between the arterial staining lesion with an iodine contrast agent and liver parenchyma that is mildly contrasted, thereby improving the lesion conspicuity (Figure 4). Alternatively, the same contrast as that obtained at 120 kVp can be obtained even if the amount of contrast medium is reduced. A monochromatic image of approximately 70 keV shows a CT value equivalent to that of a conventional CT image acquired at a tube voltage of 120 kVp. However, in a low energy monochromatic image, because the iodine CT value increases in a similar manner to that observed with the low tube voltage imaging described above, it is conceivable that the contrast of tumors and the liver can be increased even with a low contrast agent volume.

The Liver Imaging-Reporting and Data System (LI-RADS) is a comprehensive system for standardized interpretation and reporting of CT and MR liver examinations to overcome the mentioned limits and achieve congruence with HCC diagnostic criteria in at-risk populations. LI-RADS provides four major features including arterial phase hyperenhancement (APHE) and wash-out (WO) appearance. APHE and WO that are defined as a non-peripheral, visually assessed, temporal changes of enhancement in whole or in part compared to the surrounding liver parenchyma. Although, these features have a prominent role in the LI-RADS algorithm, an attempt to improve the delineation of these imaging findings by DECT is being studied [26].

DECT can improve delineation of both hyper- and hypovascular lesions by accentuating the lesion to liver parenchyma contrast. In low-keV arterial phase images, iodine within the hypervascular lesions shows higher attenuation as compared to the background liver increasing the conspicuity of the lesion (Figure 4), whereas hypovascular lesions scanned in the portovenous phase at low keV show lower attenuation as compared to the parenchyma due to a greater distribution of iodinated contrast within the normal hepatic tissue. Recently, an in vitro study performed on layer-detector ssDECT also showed higher CNR of both hyper- and hypovascular lesions on low-keV images. Irrespective of lesion and scanner type, the increased CNR at low photon energies reveals more lesions (Figure 3) with increased acuity and improved definition of margins (Figure 4). This is especially useful in assessing the extent of diffuse infiltrative masses and surgical planning [27,28,29].

Researchers suggest that the increase in the amount of iodine contrast agent administered during contrast-enhanced CT is related to the increased risk of contrast-agent-induced nephropathy [30]. It is considered that DECT is most common utilized for contrast-enhanced CT in patients with renal dysfunction [31], and for dynamic CT imaging of the hepatobiliary and pancreatic regions that require higher amounts of contrast agents. According to a prior study that used DECT imaging for hepatocellular carcinoma, it was reported that detectability improved without any image quality degradations with a monochromatic image acquired at 40 to 70 keV [32]. In addition, it is possible to reduce the beam-hardening artifact that caused deterioration of image quality and CT value errors. Virtual, single monochromatic imaging also made it possible to prevent shifting of CT values by metals, or high-concentrations of contrast agents. This implies that there is a possibility that the density information of each tissue pixel and altered lesions can be analyzed more accurately. This is useful for the evaluation of the bile ductal region and the stent lumen after endovascular aortic aneurysm repair in the abdominal region.

### 3.2. Material Decomposition

Histological evaluation by liver biopsy is the gold standard in the evaluation of hepatic fibrosis and fatty liver. However, it is difficult to conduct the evaluation repeatedly owing to the degree of invasiveness. Thus, the variability in the evaluation between pathologists and sampling errors also constitutes a problem. Quantification of fat, iron, and other moieties by CT means that the degree of liver disease can be quantitatively diagnosed by segments instead of histological diagnosis with more invasive biopsy. It is expected to be very useful for preoperative evaluation, postoperative prognosis, and prediction of therapeutic effects. For this reason, noninvasive quantitative imaging techniques have been studied in recent years. In general, magnetic resonance imaging (MRI) has been the most accurate method for assessing hepatic fibrosis and fat deposition. However, because of the high cost of examination and the length of the examination time, ultrasonography is less reproducible and technological differences among examiners are minor. CT is a commonly used imaging modality in routine clinical practice, has high reproducibility, can be used for relatively short time periods, has a low cost, and performs quantitative evaluations of diffuse liver disease and follow up examinations.

**Figure 5:**
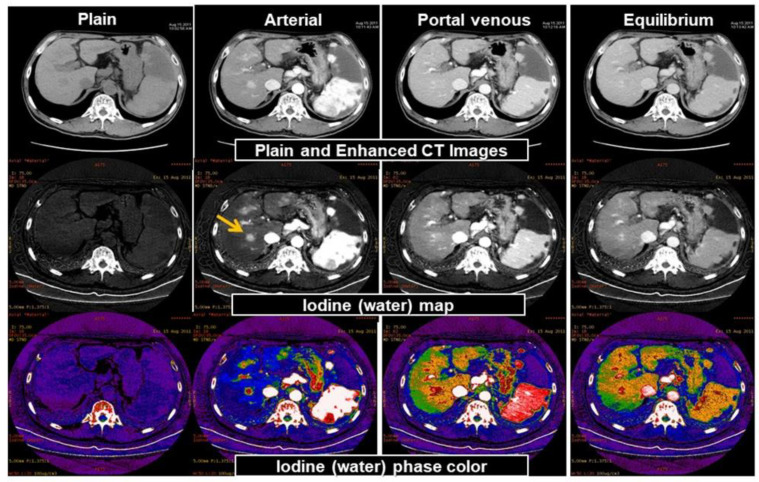
Hepatocellular carcinoma. Dynamic CT (upper panel), iodine density image (middle panel), iodine density color image (lower panel). Hepatocellular carcinoma deeply stained in the arterial phase of dynamic CT is recognized. Density iodine imaging clearly shows tumor staining. Density iodine color imaging shows tumor deeply stained by arteries and iodine distribution in the hepatic parenchyma deeply stained by portal blood flow.

In conventional CT, it is difficult to visually distinguish a contrast agent from calcifications because both have similar CT values. For example, evaluation of a blood vessel lumen with severe calcification was difficult in the past, but with DECT, it is possible to decompose materials such as calcium, iodine (contrast agent), and fat. The changes in the CT values owing to energy differences show a pattern that is unique to each substance. For example, different curves exist for iodine and calcium (Figure 2). The factor that causes the most prominent effects on the spectral characteristic is the atomic number *Z*. Therefore, substances with sufficiently large differences in their effective atomic numbers can be discriminated via DECT imaging. The Z values of hydrogen, oxygen, carbon, and nitrogen that primarily comprise the human body are as low as 1, 8, 6, and 7, respectively. It is easy to discriminate between iodine (*Z* = 53) and human tissues. Alternatively, bone and calcification consisting of Ca (*Z* = 20) are therefore easy to distinguish from other human tissues that are composed only of hydrogen and carbon. By using this principle, it is possible to create an iodine density image (Iodine map shown in Figure 5, calcium density image, water density image, and fat density image). Furthermore, by determining the iodine/water density distribution and the water/fat density distribution, it is also possible to quantify iodine as well as fat and water density (mg/cm^3^) in regions-of-interest (ROIs) by marking the ROIs. Its use for clinical applications in the liver has been reported for quantitative assessments of fat deposition and steatosis in fatty liver and nonalcoholic steatohepatitis (NASH) instead of tissue diagnosis that is achieved via highly invasive biopsies. Additionally, it can be useful in methods that use iodine density of lipiodol as an indicator for drug delivery to tumors during transcatheter arterial chemoembolization (TACE) using lipiodol [33].

### 3.3. Virtual Unenhanced CT

Conversely, by acquiring a water density image, a virtual unenhanced (VUE) CT images of the liver created by accurately removing the concentration of the contrast agent from the contrast CT, can be obtained. If simple CT is omitted and only contrast CT is performed with DECT, and if simple CT can be substituted by the resulting virtual non-contrast CT, this is expected to lead to a reduction in exposure to radiation [34]. In addition, in instances where contrast alone is difficult to distinguish a contrasted hepatocellular carcinoma from a hemorrhagic cyst that exhibits high concentration, examining the concentration by virtual non-contrast CT would make the distinction possible.

### 3.4. Limitations

It is necessary to show some shortcomings of DECT before investment in scanner and interpretation of images. Technical limitations with respect to hardware include limited FOV and reduced spectral separation, depending on vendor or scanner. Furthermore, in obese patients, photon starvation in the low-voltage acquisition causes increased image noise and limits interpretation of DECT. Photon starvation is especially prominent in the region of the diaphragm, leading to pseudolesions in DECT at the hepatic dome. Software challenges include lack of enough studies comparing inter-vendor and inter-scanner variability of attenuation values on virtual VUE and iodine concentration on material-specific iodine images. Managerial limitations include workflow challenges due to increased time needed for image reconstructions, need for larger data storage capacity, high cost of scanners, and lack of reimbursement for DECT applications [34].

## 4. Evaluation of Hepatic Steatosis Using DECT

**Figure 6:**
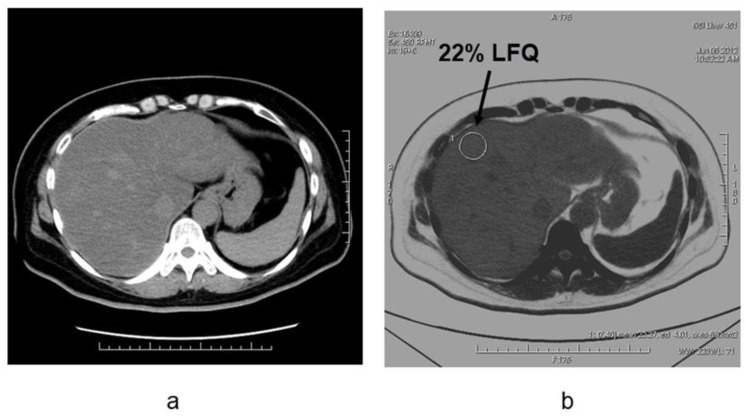
Liver fat measurements by using dual-energy CT in the patient with NAFLD (non-alcoholic fatty liver disease)(**a**) Noncontrast CT image and (**b**) fat density image in patients with nonalcoholic fatty liver disease. The degree of fat deposition was quantifiable on the fat density images, and the quantitative assessment of liver tissue and fat in this case was 22% liver-fat quantification.

Conventionally available methods include MR spectroscopy (MRS), the dual-echo MRI method, CT measurements, and visual evaluation via ultrasonic tests. Among these, MRS (magnetic resonance spectroscopy) has yielded the highest fat content [35]. Conversely, new quantitative methods have been developed for ultrasound tests, MRI, and CT. According to a meta-analysis [36], the detectability of moderate/severe (20–30%) fatty liver using B-mode ultrasound-based qualitative assessment had a sensitivity of 84.8% (95% CI: 79.5 to 88.9) and a specificity of 93.6% (87.2 to 97.0). Chemical shift imaging is said to improve quantitativeness by correcting the influence of T1 value, T2* attenuation, variation in precession frequency of fat protons, etc. [37,38]. A few studies have measured the amount of fat obtained through T1-independent, T2*-corrected chemical shift imaging with multi-peak fat spectral modeling relative to the MRS-proton density fat fraction (MRS-PDFF) measured using MRS, as MRI-PDFF [38]. Although it is recognized that the MR test is the most accurate for fat determination among each of the imaging modalities, there are variations in the method and it is difficult to construct coherent evidence at the present time [39]. As fibrosis occurs due to hepatic injury, the amount of fat decreases; thus, low fat quantity detected on imaging does not guarantee a milder case and this is applicable for not only MRI, but also CT and ultrasound tests. In CT, the semiquantitative evaluation using X-ray absorption values (HUs) has been extensively known. Although it is convenient to conduct these evaluations based on the CT value of the liver parenchyma, the CT value may not necessarily be consistent among various CT scanners. Therefore, the evaluation with the use of a ratio or difference in CT values between liver and spleen should be preferred. Park et al. [40] argued that the difference in liver to spleen CT values in conventional CT had a reference range between 1–18 HU in nonfatty livers, where 1 HU (or less) is indicative of a fatty liver. A few recent reports have suggested that with DECT imaging—that is becoming widespread in recent years—it is possible to quantify the amount of liver fat in contrast CT based on material decomposition that helps distinguish two or more substances [41,42,43,44,45] (Figure 6).

Hyodo et al. reported that the area under the receiver operating characteristic (ROC) curve of DECT was 0.88 (95% confidence interval 0.74–0.98) in the detection of fatty liver with more than 5% fat in needle biopsy tissue. This was equivalent to MRS (area under the ROC curve 0.89) (95% confidence interval 0.724–1) [41]. This study also suggested that high-precision fat quantification is possible in postcontrast images. Hyodo et al. also reported that fat was underestimated by the presence of iron deposited in the liver parenchyma [42], while the phantom study conducted by Fishcer et al. showed that accurate fat quantitation was possible even in the presence of iron [43].

Evaluation of active inflammation in addition to fat is crucial for the early diagnosis and staging of NASH. Because it is difficult to evaluate inflammation with imaging studies with CT alone, the future challenge will involve accurate assessments in conjunction with other examinations.

## 5. Estimation of Hepatic Fibrosis Using DECT

Given the degree of hepatic fibrosis correlates with carcinogenesis and prognosis, accurate evaluation is important in determining the clinical course and in predicting the prognosis. Non-invasive diagnosis and staging of hepatic fibrosis are important for evaluating disease progression in patients with chronic liver diseases. Serum markers of liver fibrosis are widely available, but their results are variable. Ultrasound elastography is used for grading hepatic fibrosis, but it is operator dependent [46,47,48]. Several magnetic resonance (MR) imaging sequences, such as perfusion-weighted MR imaging, MR spectroscopy, MR elastography, and dynamic contrast-enhanced MR have the potential to provide quantitative information with variable diagnostic accuracy in the staging of liver fibrosis. DWI provides noninvasive quantification of water diffusion and can be used for in vivo quantification of the combined effects of capillary perfusion and diffusion. There are limited data on correlation between ADC and degree of fibrosis and other histopathological findings (inflammation, steatosis, iron content, necrosis, cholestasis) in the liver [49]. Besheer et al. reported that the ADC value was decreased from controls (F0) to patients with early fibrosis and those with late fibrosis. Combined ADC and miR-200b revealed the best result for differentiating early from late fibrosis and offer an alternative surrogate non-invasive diagnostic tool for diagnosis and staging of hepatic fibrosis in patients with chronic hepatitis C [50].

There are insufficient differences in the mass attenuation coefficients between fibrous and normal liver tissue, and it is difficult to quantify hepatic fibrosis by SECT. However, it is possible to indirectly estimate the degree of fibrosis with contrast media as a marker. When fibrosis occurs in liver tissue, deposition of collagen fibers expands the extracellular space. Histologically, the degree of hepatic fibrosis is strongly correlated with the volume of the extracellular space (ECV), and the quantification of ECV can be used to estimate the degree of hepatic fibrosis [51]. The equilibrium phase of contrast-enhanced CT is used for the determination of ECV in liver tissue. Given that the administered contrast medium diffuses from the intravascular space into the extravascular space and equilibrates over time, the concentration of contrast medium in the intravascular and extravascular spaces is considered to be equal during the equilibrium phase of contrast-enhanced CT. Therefore, by normalizing the contrast enhancement in the liver parenchyma in the equilibrium phase by the contrast enhancement in the blood pool, such as in the aorta, the percentage of ECV fraction in the liver tissue (ECV fraction) can be measured, and the ECV fraction can thus be calculated using the formula: (enhancement in the liver)/(enhancement in the aorta) × (1 − hematocrit).

In SECT, the difference between pre- and postcontrast CT values is used to quantify the ECV fraction or to estimate hepatic fibrosis. The time from the injection of contrast medium to the imaging of the equilibrium phase varied between 3 and 30 min [52,53,54,55]. Yoon et al. and Guo et al. reported that the ECV fraction calculated using the equilibrium phase 3 min after contrast was significantly correlated with the METAVIR score (correlation coefficient *ρ* = 0.493 and *ρ* = 0.468, respectively) [54,55]. In addition, the ECV fraction calculated using nonlinear nonrigid alignment (SURE subtraction) from the equilibrium phase 4 min after contrast enhancement, and the pre-contrast images, showed a strong correlation with the METAVIR score (*ρ* = 0.71) [56]. In DECT, a more quantitative ECV fraction can be estimated based on the use of the difference in iodine density between pre- and postcontrast images. Sofue et al. reported that the ECV fraction measured using iodine density images in the equilibrium phase 3 min after contrast was significantly correlated with the METAVIR score (*ρ* = 0.67) [57]. The effect of hepatic active inflammation and edema on the quantitation of ECV is also unclear. These verifications and further improvements in the quantitative accuracy seem to be future problems.

## 6. Potential Future Directions

DECT is a relatively new technological development. It has been shown to be potentially useful in a wide variety of diseases. Recent advances by manufacturers decreased the impact of such techniques on radiation dose. Yu et al. [58] found that DECT used in adult imaging can produce a set of images for routine diagnostic interpretation that are of similar or improved quality compared with conventional, single-energy, 120 kVp scans with the same level of radiation exposure. Future developments for DECT may include dose reductions by automated tube current modulation and user-modifiable iterative reconstruction. More accurate, or quantitative/semiquantitative approaches used to identify enhancement with iodinated contrast would have significant advantages over conventional CT density numbers (HU) derived by polychromatic beam imaging as these are not absolute and can vary by scanner, reconstruction technique, patient size, and the X-ray tube potential [59]. Material-density images allow the quantitative or semiquantitative estimations of the amount of iodine. These may thus be helpful in providing additional characteristics for identifying tumor treatment response.

DECT can detect and differentiate iodine contrast and calcium based on their HU curves. Tran et al. [60] has indicated that the accuracy depends on the CT density of tissue and is limited when the CT attenuation is low. Future improvements in this area are expected. The possibility of even more advanced techniques, such as photon counting and better discernment of even lower energies, may improve characterization of materials within tissues, and will thus offer additional benefits for oncological imaging.

## 7. Conclusions

Use of DECT allows the distinction and quantification of unique substances, such as iodine, calcium, fat, and iron. This task could not be performed with conventional CT. This implies that data related to tissues can be evaluated in addition to the information derived from conventional CT examinations, such as morphology and blood flow information. Furthermore, we expect to achieve reductions in radiation exposure and in the amount of contrast agent used by performing CT with low exposure and increased contrast volumes using virtual monochromatic X-ray imaging. To achieve application of these techniques in daily clinical use, additional research and development is necessary. Accordingly, many research studies are ongoing.
